# Gut Symbionts Lactobacillus reuteri R2lc and 2010 Encode a Polyketide Synthase Cluster That Activates the Mammalian Aryl Hydrocarbon Receptor

**DOI:** 10.1128/AEM.01661-18

**Published:** 2019-05-02

**Authors:** Mustafa Özçam, Restituto Tocmo, Jee-Hwan Oh, Amin Afrazi, Joshua D. Mezrich, Stefan Roos, Jan Claesen, Jan-Peter van Pijkeren

**Affiliations:** a Department of Food Science, University of Wisconsin—Madison, Madison, Wisconsin, USA; b Department of Surgery, University of Wisconsin—Madison, Madison, Wisconsin, USA; c Department of Molecular Sciences, Swedish University of Agricultural Sciences, Uppsala, Sweden; d Department of Cellular and Molecular Medicine, Lerner Research Institute, Cleveland Clinic, Cleveland, Ohio, USA; University of Georgia

**Keywords:** *Lactobacillus reuteri*, probiotic, aryl hydrocarbon receptor, biosynthetic gene cluster, gut symbiont, pigment, polyketides, secondary metabolism

## Abstract

A mechanistic understanding of microbe-host interactions is critical to developing therapeutic strategies for targeted modulation of the host immune system. Different members of the gut symbiont species Lactobacillus reuteri modulate host health by, for example, reduction of intestinal inflammation. Previously, it was shown that L. reuteri activates the aryl hydrocarbon receptor (AhR), a ligand-activated transcription factor that plays an important role in the mucosal immune system, by the production of tryptophan catabolites. Here, we identified a novel pathway by which L. reuteri activates AhR, which is independent of tryptophan metabolism. We screened a library of 36 L. reuteri strains and determined that R2lc and 2010, strains with a pigmented phenotype, are potent AhR activators. By whole-genome sequencing and comparative genomics, we identified genes unique to R2lc and 2010. Our analyses demonstrated that R2lc harbors two genetically distinct polyketide synthase (PKS) clusters, functionally unknown (*fun*) and *pks*, each carried by a multicopy plasmid. Inactivation of *pks*, but not *fun*, abolished the ability of R2lc to activate AhR. L. reuteri 2010 has a gene cluster homologous to the *pks* cluster in R2lc with an identical gene organization, which is also responsible for AhR activation. In conclusion, we identified a novel PKS pathway in L. reuteri R2lc and 2010 that is responsible for AhR activation.

**IMPORTANCE** Temporary changes in the composition of the microbiota, for example, by oral administration of probiotics, can modulate the host immune system. However, the underlying mechanisms by which probiotics interact with the host are often unknown. Here, we show that Lactobacillus reuteri R2lc and 2010 harbor an orthologous PKS gene cluster that activates the aryl hydrocarbon receptor (AhR). AhR is a ligand-activated transcription factor that plays a key role in a variety of diseases, including amelioration of intestinal inflammation. Understanding the mechanism by which a bacterium modulates the immune system is critical for applying rational selection strategies for probiotic supplementation. Finally, heterologous and/or optimized expression of PKS is a logical next step toward the development of next-generation probiotics to prevent and treat disease.

## Abstract (precis)

Temporary changes in the composition of the microbiota, for example, by oral administration of probiotics, can modulate the host immune system. However, the underlying mechanisms by which probiotics interact with the host are often unknown. Here, we show that Lactobacillus reuteri R2lc and 2010 harbor an orthologous PKS gene cluster that activates the aryl hydrocarbon receptor (AhR). AhR is a ligand-activated transcription factor that plays a key role in a variety of diseases, including amelioration of intestinal inflammation. Understanding the mechanism by which a bacterium modulates the immune system is critical for applying rational selection strategies for probiotic supplementation. Finally, heterologous and/or optimized expression of PKS is a logical next step toward the development of next-generation probiotics to prevent and treat disease.

## INTRODUCTION

The mammalian intestinal tract contains trillions of bacteria, which collectively contribute to our well-being (1). Metagenomic studies suggest that microbial metabolites play a central role in microbe-host interactions, including regulation of the host immune system (2). Therefore, a mechanistic understanding of metabolite-mediated microbe-host interactions is critical for developing therapeutic strategies for targeted modulation of the host immune system.

Polyketide synthases (PKS) and nonribosomal peptide synthases (NRPS) are secondary metabolites produced by biosynthetic gene clusters (BCGs) that assemble simple molecules, such as acetyl-CoA, into complex metabolites, some of which (i.e., erythromycin) are important to the pharmaceutical industries (3). Many medically important biosynthetic gene clusters were identified from soil and marine microbes, including the antitumor polyketide onnamide (4) and the immunosuppressant rapamycin (5). Although PKS gene clusters have previously been identified in the human gut microbiome (6), our understanding of their role in immunomodulation is limited. One of the few examples describing the role of PKS clusters in immunomodulation is the *pks* island cluster in Escherichia coli Nissle 1917. Inactivation of the PKS island in Nissle 1917 results in a loss of anti-inflammatory function in the adoptive T-cell transfer model of inflammation (7).

PKS and NRPS clusters have been identified in other lactic acid bacteria (LAB). For example, the oral pathogen Streptococcus mutans encodes an NRPS/PKS system that provides the organism with an advantage in multispecies biofilms in dental caries (8). LAB that have been associated with food, like Lactococcus lactis KF147 (mung bean sprouts) and the sourdough isolates Lactobacillus reuteri LTH2584, TMW1.106, TMW1.112, and TMW1.656, also encode NRPS/PKS systems (9, 10). It was suggested that the NRPS/PKS cluster in L. reuteri contributes to persistence in sourdough (10). Thus, the identified NRPS/PKS clusters in those LAB appear to be advantageous to the organisms’ ability to thrive in their niches.

The host can recognize microbial metabolites via different pathways, including via pattern recognition receptors and the aryl hydrocarbon receptor (AhR) (11, 12). Pigmented virulence factors like phenazines and naphthoquinone phthiocol are detected by the host immune system and degraded through AhR-mediated cytokine and chemokine production (12). AhR is a ligand-activated transcription factor that recognizes environmental pollutants, dietary compounds (i.e., glucobrassicin and flavonoids), and microbial-derived secondary metabolites (i.e., indole-3-carbinol). Upon ligand binding, AhR translocates into the nucleus to induce target gene expressions. The role of AhR has been extensively studied in relation to metabolism of environmental toxins, but the focus has recently shifted to its role in modulation of the adaptive and innate immune system. The activation of AhR, for example, is important for the production of interleukin-22 (IL-22), which enhances the innate immune response by inducing the production of antimicrobial peptides (Reg3 lectins) to fight off intestinal pathogens and to protect intestinal tissues from inflammation damage by inducing tight junction proteins (13, 14).

Recently, different strains of L. reuteri have been shown to activate AhR and modulate the immune system through tryptophan (Trp) metabolism (15–17). For example, L. reuteri 100-23 metabolizes dietary tryptophan into bioactive indole derivatives, which subsequently activates AhR. AhR activation by L. reuteri 100-23 increases *Il22* expression in the intestine, which in turn provides protection from *Candida* infection in mice that could not take up tryptophan (13). In mammals, 99% of dietary tryptophan is taken up by the host (18), thereby limiting tryptophan availability to the microbiota to produce AhR ligands. Therefore, identification of specific gut microbes, including L. reuteri strains, that activate AhR independent of tryptophan metabolism is important for developing AhR-mediated biotherapeutic strategies to target intestinal diseases.

Here, we screened an L. reuteri library of 36 strains with different host origins for their ability to activate AhR. We employed an *in vitro* screening method and identified that L. reuteri R2lc and 2010 are potent AhR activator strains. By whole-genome sequencing, comparative genomics, and targeted gene inactivation, we discovered that an orthologous PKS cluster is responsible for the AhR activation phenotype in L. reuteri R2lc and 2010.

## RESULTS

### L. reuteri R2lc and 2010 are potent AhR activators.

**FIG 1:**
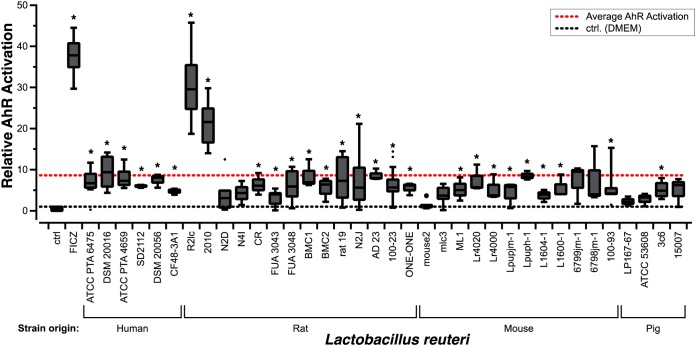
Assessment of Lactobacillus reuteri AhR activation potential. L. reuteri AhR activation is strain specific. Data are represented as fold changes relative to the DMEM control (ctrl). The positive control is DMEM supplemented with 500 nM the AhR ligand 6-formylindolo[3,2-b]carbazole (FICZ). Data are presented as box-and-whisker plots. The whiskers represent the maximum and minimal values, and the lower, middle, and upper lines of the box represent first quartile, median, and third quartile, respectively. Open circles represent suspected outliers, which are data points that are 1.5 times below the first quartile or 1.5 times above the third quartile. Closed circles represent outliers, which are 3 times the value of the first or third quartile. Data represent averages of results from 3 independent experiments. Statistical significance between strains and the DMEM control was determined by the one-sample *t* test. Asterisks show statistical significance (*P* < 0.05).

To determine the capacity of L. reuteri to activate AhR, we exploited an *in vitro* reporter cell line, H1L6.1c3, in which luminescence levels are positively correlated with AhR activation (19). We screened cell-free supernatants of 36 L. reuteri strains of human, pig, and rodent origins (Table 1). Compared to the medium control, 27 out of 36 strains activated AhR (*P < *0.05) (Fig. 1). Importantly, the cell-free bacterial supernatants had no effect on cell viability (see Fig. S1 in the supplemental material). We found that two pigmented strains, R2lc and 2010, were considerably more potent in their ability to activate AhR (30.2-fold ± 8.3-fold and 20.9-fold ± 5.6-fold, respectively) than other L. reuteri strains (mean activation, 8.6-fold). To understand the underlying mechanism by which R2lc and 2010 activate AhR, we focused first on L. reuteri R2lc for several reasons. First, R2lc has been safely consumed by humans (20, 21), which, to some extent, paves the way to apply this strain in future human trials. Second, several research groups demonstrated that R2lc reduces intestinal inflammation in different inflammation disease models (22–26), reduces acute liver injury (27), and reduces bacteremia (28).

**TABLE 1:** Bacterial strains used in this study^*a*^

Species	Strain	VPL identifier	Origin or description	Source and/or reference
Lactobacillus reuteri	CF48-3A1	VPL1086	Human	BioGaia AB, JGI 2502171173
Lactobacillus reuteri	SD2112	VPL1013	Human	BioGaia AB
Lactobacillus reuteri	ATCC PTA 6475	VPL1014	Human	BioGaia AB
Lactobacillus reuteri	DSM 20016	VPL1046	Human	JGI 640427118
Lactobacillus reuteri	ATCC PTA 4659	VPL1031	Human	BioGaia AB
Lactobacillus reuteri	DSM20056	VPL1061	Human	JGI 642555135
Lactobacillus reuteri	R2lc	VPL1053	Rat	Siv Ahrné, JGI 2716884882
Lactobacillus reuteri	2010	VPL1054	Rat	BioGaia AB, JGI 2710724192
Lactobacillus reuteri	N2D	VPL1067	Rat	Siv Ahrné
Lactobacillus reuteri	FUA3043	VPL1062	Rat	49
Lactobacillus reuteri	FUA3048	VPL1063	Rat	49
Lactobacillus reuteri	BMC1	VPL1093	Rat	Stafan Roos
Lactobacillus reuteri	BMC2	VPL1057	Rat	Stefan Roos
Lactobacillus reuteri	100-23	VPL1049	Rat	JGI 2500069000
Lactobacillus reuteri	CR	VPL1059	Rat	49
Lactobacillus reuteri	Rat 19	VPL1069	Rat	49
Lactobacillus reuteri	AD 23	VPL1048	Rat	49
Lactobacillus reuteri	N2J	VPL1052	Rat	Siv Ahrné
Lactobacillus reuteri	N4I	VPL1063	Rat	49
Lactobacillus reuteri	mouse 2	VPL1070	Mouse	49
Lactobacillus reuteri	one-one	VPL1060	Mouse	49
Lactobacillus reuteri	6799jm-1	VPL1051	Mouse	49
Lactobacillus reuteri	L1600-1	VPL1064	Mouse	49
Lactobacillus reuteri	100-93	VPL1047	Mouse	49
Lactobacillus reuteri	Lr4020	VPL1072	Mouse	49
Lactobacillus reuteri	6798jm-1	VPL1055	Mouse	49
Lactobacillus reuteri	mlc3	VPL1050	Mouse	JGI 2506381016
Lactobacillus reuteri	Lpuph-1	VPL1056	Mouse	JGI 2506381017
Lactobacillus reuteri	Lr4000	VPL1071	Mouse	BioGaia AB
Lactobacillus reuteri	ML1	VPL1058	Mouse	49
Lactobacillus reuteri	L1604-1	VPL1066	Mouse	49
Lactobacillus reuteri	lpupjm1	VPL1065	Mouse	49
Lactobacillus reuteri	3c6	VPL1083	Pig	JGI 2599185333
Lactobacillus reuteri	Lp167-67	VPL1085	Pig	BioGaia AB, JGI 2599185361
Lactobacillus reuteri	I5007	VPL1082	Pig	JGI 2554235423
Lactobacillus reuteri	ATCC 53608	VPL1090	Pig	BioGaia AB, EMBL accession no. LN906634
Lactobacillus reuteri	TMW1.112	VPL1089	Sourdough	JGI 2534682347
Lactobacillus reuteri	TMW1.656	VPL1088	Sourdough	JGI 2534682350
Escherichia coli	EC1000	VPL1009	*In trans* RepA provider; Kan^r^	50
Escherichia coli	VPL3002	VPL3002	EC1000 harboring pVPL3002; Em^r^	29
Lactobacillus reuteri	ATCC PTA 6475	VPL3025	Harboring plasmid pJP042	44
Lactobacillus reuteri	R2lc *ΔaraT*	VPL4192	Deletion of *araT* gene in R2lc	29
Lactobacillus reuteri	R2lc *Δfun*	VPL4208	Deletion of genes *funE*, *funF*, and *funG* in R2lc	This study
Lactobacillus reuteri	R2lc *Δpks*::Cm	VPL4209	Replacement of genes *pksG*, *pksH*, and *pksI* with the gene encoding chloramphenicol resistance	This study
Lactobacillus reuteri	2010 *Δpks*	VPL4183	Deletion of genes *pksG*, *pksH*, and *pksI* in 2010	This study

a VPL, van Pijkeren Laboratory strain identification number. Further information on the listed Joint Genome Institute (JGI) source sequences can be found at the JGI genome portal (http://genome.jgi.doe.gov).

### L. reuteri R2lc and 2010 produce bright orange pigment.

**FIG 2:**
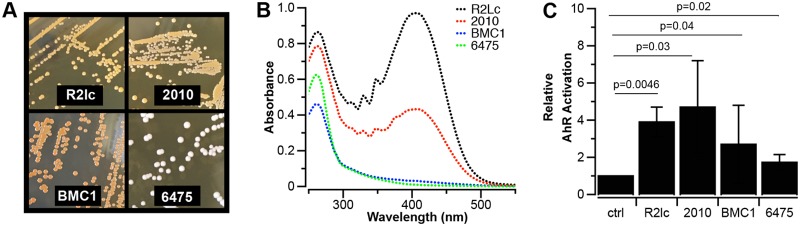
Orange pigments extracted from R2lc, 2010, and BMC1 are not strong AhR ligands. (A) R2lc, 2010, and BMC1 produce an orange pigment, while most strains, like 6475, are opaque. (B) R2lc and 2010 have λ_max_ values of 405 to 409 nm. BMC1 and 6475 did not absorb at this region. (C) Methanol extracts from R2lc, 2010, and BMC1 do not show strong AhR activation. Data shown represent averages from three biological replicates, and error bars represent standard deviations. Statistical significance between extracts and the negative control was determined by the one-sample *t* test. AhR activation was not different between strains (*P* > 0.05). Statistical significance was determined by Student’s *t* test (a *P* value of <0.05 was considered significant).

Three out of 36 strains, R2lc, 2010, and BMC1, are pigmented (Fig. 2A). Although BMC1 did not show a strong AhR activation phenotype, we reasoned that this might be due to differences in the chemical structures of the pigments. To analyze this, we first performed methanol extraction, followed by UV-visible spectroscopy. We identified that both R2lc and 2010 have an absorption wavelength maximum (λ_max_) of 405 to 409 nm, while this peak was absent in BMC1 and 6475 (Fig. 2B). These data show that the pigments of L. reuteri R2lc and 2010 have similar properties, while the pigment of BMC1 is distinctly different. Second, we tested all extracts for their ability to activate AhR. We found that all extracts, including that of a nonpigmented strain (6475), increased AhR activation (Fig. 2C); however, each of the extracts was much less potent than the cell-free supernatants of R2lc and 2010. Together, these data suggested that the orange pigments produced by R2lc and 2010 are not the main drivers of the strong AhR activation phenotype.

### L. reuteri R2lc AraT-mediated tryptophan metabolism is not the main driver of AhR activation.

**FIG 3:**
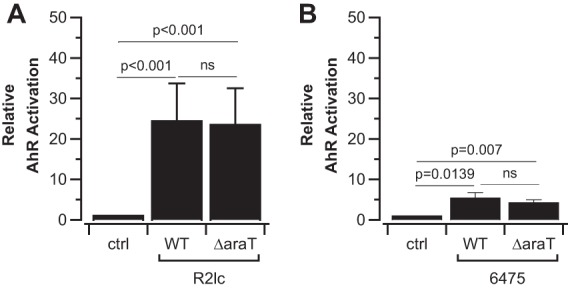
R2lc activates AhR independent of AraT. (A) AraT is not the main driver of AhR activation by R2lc. (B) Deletion of the *araT* gene in 6475 does not significantly change the AhR activation level in L. reuteri 6475. Data shown represent averages of results from three biological replicates, and error bars represent standard deviations. Statistical significance between strains and the negative control was determined by the one-sample *t* test. Statistical significance between strains was determined with Student’s *t* test. ns, not significant; WT, wild type.

Previously, several groups demonstrated that L. reuteri activates AhR via indole molecules acquired by aromatic amino acid amino transferase (AraT)-mediated tryptophan metabolism (15, 17). To determine to what extent L. reuteri R2lc AraT plays a role in AhR activation, we used a derivative of R2lc in which *araT* was deleted (R2lc Δ*araT*) (29). In our experimental setup, R2lc Δ*araT* did not reduce the AhR-activating potential of L. reuteri R2lc (Fig. 3A), suggesting that another mechanism is driving AhR activation in R2lc. To test if AraT, which is conserved in L. reuteri, contributes to the basal level of AhR activation, we inactivated *araT* in L. reuteri 6475, a strain that activates AhR at a reduced level compared to R2lc. L. reuteri 6475 Δ*araT* displayed a nonsignificant 20% reduction in AhR activation compared to the wild type (Fig. 3B). Thus, at least in our model system, AraT marginally impacts AhR activation of L. reuteri 6475 and is not a major driver by which L. reuteri R2lc activates AhR.

### L. reuteri R2lc harbors two multicopy plasmids.

**FIG 4:**
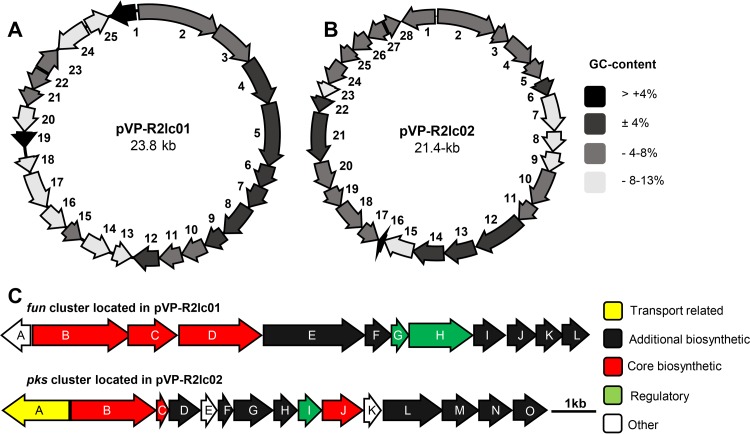
Overview of the pVP-R2lc01 and pVP-R2lc02 plasmids and their predicted PKS clusters. (A) The 23.8-kb pVP-R2lc01 plasmid contains 25 ORFs (indicated by arrows), including the *fun* cluster (ORFs 1 to 12). (B) The 21.4-kb pVP-R2lc02 plasmid contains 28 ORFs and carries the *pks* cluster (ORFs 1 to 15). Differences in GC contents of each gene (compared to the chromosomal GC content) are represented with different colors, as shown on the right. (C) The *fun* cluster (top) spans 13.4 kb containing 12 ORFs, and the *pks* cluster (bottom) spans 11.3 kb containing 15 ORFs. Transport-related, additional biosynthetic, core biosynthetic, regulatory, and other genes are represented (for gene annotations, see Tables 2 and 3).

To identify the AhR activation pathway in L. reuteri R2lc, our strategy was to first identify genes unique to R2lc, followed by gene inactivation to assess the phenotype. To this end, we compared the draft genome of L. reuteri R2lc with the genomes of 10 L. reuteri strains that we determined have a relatively low AhR activation potential. We identified 79 putative genes unique to L. reuteri R2lc (Table S2). Interestingly, within this pool of unique genes, several genes putatively encode proteins involved in polyketide production and plasmid replication, which were distributed over five contigs. By primer walking and gap closure, we identified a 23.8-kb plasmid and a 21.4-kb plasmid, here referred to as pVP-R2lc01 and pVP-R2lc02, respectively. Plasmid pVP-R2lc01 contains 25 open reading frames (ORFs) (Fig. 4A and Table 2), while pVP-R2lc02 contains 28 ORFs (Fig. 4B and Table 3). The pVP-R2lc01 and pVP-R2lc02 plasmids have similar GC contents (32.4% and 32.1%, respectively), which are lower than the predicted chromosomal GC content (38.5%) (Table 4). By quantitative real-time PCR, we determined that, relative to the chromosome, pVP-R2lc01 and pVP-R2lc-02 are present at 3.75 ± 1.14 and 3.82 ± 0.62 copies, respectively.

**TABLE 2:** Predicted annotations of genes in pVP-R2lc01^*a*^

ORF (gene)	Locus tag	Predicted function	Length (amino acids)
1 (*funA*)	C5O77_00105	Biotin-(acetyl-CoA-carboxylase) ligase	250
2 (*funB*)	C5O77_00110	Beta-ketoacyl synthase domain	798
3 (*funC*)	C5O77_00115	8-Amino-7-oxononanoate synthase	399
4 (*funD*)	C5O77_00120	Hypothetical protein (acyl carrier protein)	663
5 (*funE*)	C5O77_00125	Hypothetical protein [NAD(P)-dependent dehydrogenase, short-chain alcohol dehydrogenase family]	847
6 (*funF*)	C5O77_00130	Hypothetical protein (phosphopantetheinyl transferase)	228
7 (*funG*)	C5O77_00135	Hypothetical protein (transcriptional regulator, TetR family)	203
8 (*funH*)	C5O77_00140	Acetyl-CoA carboxylase biotin carboxyl carrier protein subunit	135
9 (*funI*)	C5O77_00145	Acetyl-CoA carboxylase biotin carboxylase subunit	447
10 (*funJ*)	C5O77_00150	Acetyl-CoA carboxylase carboxyl transferase subunit beta	270
11 (*funK*)	C5O77_00155	Acetyl-CoA carboxylase carboxyl transferase subunit alpha	249
12 (*funL*)	C5O77_00160	NAD(P)H dehydrogenase	194
13	C5O77_00165	Replication-associated protein RepC	126
14	C5O77_00170	ATPase	292
15	C5O77_00050	Hypothetical protein	106
16	C5O77_00055	Hypothetical protein	219
17	C5O77_00060	Hypothetical protein (relaxase/mobilization nuclease domain-containing protein)	461
18	C5O77_00065	Mobilization protein (MobC)	124
19	C5O77_00070	Hypothetical protein	78
20	C5O77_00075	Hypothetical protein	96
21	C5O77_00080	XRE family transcriptional regulator	100
22	C5O77_00085	Hypothetical protein (RelE toxin of the RelE/RelB toxin-antitoxin system)	129
23	C5O77_00090	Hypothetical protein	122
24	C5O77_00095	Site-specific integrase	195
25	C5O77_00100	Hypothetical protein	95

a Annotations were obtained from the JGI-IMG automated annotation pipeline.

**TABLE 3:** Predicted annotations of genes in pVP-R2lc02^*a*^

ORF (gene)	NCBI locus tag	Predicted function^*b*^	Length (amino acids)
1 (*pksA*)	C5O77_01185	MFS transporter	549
2 (*pksB*)	C5O77_01050	3-Oxoacyl-ACP synthase	688
3 (*pksC*)	C5O77_01055	Acyl carrier protein	90
4 (*pksD*)	C5O77_01060	Hypothetical protein (acetyl-CoA reductase 3-oxoacyl-[acyl carrier protein] reductase)	239
5 (*pksE*)	C5O77_01065	Hypothetical protein	109
6 (*pksF*)	C5O77_01070	Hypothetical protein (3-hydroxyacyl-[acyl carrier protein] dehydratase)	111
7 (*pksG*)	C5O77_01075	Hypothetical protein (glyoxylase, beta-lactamase superfamily II)	298
8 (*pksH*)	C5O77_01080	Hypothetical protein (4′-phosphopantetheinyl transferase superfamily protein)	178
9 (*pksI*)	C5O77_01085	PadR family transcriptional regulator	177
10 (*pksJ*)	C5O77_01090	(Acyl carrier protein) *S*-malonyl transferase	317
11 (*pksK*)	C5O77_01095	Acetyl-CoA carboxylase, biotin carboxyl carrier protein	142
12 (*pksL*)	C5O77_01100	Acetyl-CoA carboxylase, biotin carboxylase subunit	459
13 (*pksM*)	C5O77_01105	Acetyl-CoA carboxylase carboxyl transferase subunit beta	279
14 (*pksN*)	C5O77_01110	Acetyl-CoA carboxylase carboxyl transferase subunit alpha	257
15 (*pksO*)	C5O77_01115	Biotin-(acetyl-CoA carboxylase) ligase	262
16	C5O77_01120	tRNA_Met_CAT	
17	C5O77_01125	Hypothetical protein	104
18	C5O77_01130	ATPase	272
19	C5O77_01135	Hypothetical protein	95
20	C5O77_01140	Hypothetical protein	230
21	C5O77_01145	Hypothetical protein (relaxase/mobilization nuclease domain-containing protein)	470
22	C5O77_01150	Hypothetical protein (mobilization protein C)	124
23	C5O77_01155	Hypothetical protein	78
24	C5O77_01160	Hypothetical protein	152
25	C5O77_01165	XRE family transcriptional regulator	91
26	C5O77_01170	Type II toxin-antitoxin system RelE/ParE family toxin	120
27	C5O77_01175	Site-specific integrase	195
28	C5O77_01180	Hypothetical protein	92

a Annotations were obtained from the JGI-IMG automated annotation pipeline. MFS, major facilitator superfamily.

b Gene names were obtained from JGI-IMG annotation pipeline results. If there is another name for a specific gene, it is provided in parentheses or brackets.

**TABLE 4:** Lengths, percent G+C contents, and accession numbers for draft genomes, plasmids, and PKS clusters

Strain	Draft genome				Plasmid or contig			PKS cluster		
	Predicted length (bp)	G+C content (%)	NCBI accession no.	No. of contigs	Name	Length (bp)	G+C content (%)	Name	Length (bp)	G+C content (%)
L. reuteri R2lc	2,084,790	38.45	PTLS00000000	58	pVP-R2lc01	23,739	32.42	*fun*	13,356	30.12
					pVP-R2lc02	21,446	32.12	*pks*	12,091	31.85

L. reuteri 2010	2,214,494	38.52	PUXG00000000	38	Contig 27	69,047	35.57	*pks*	12,247	30.97

### *In silico* analyses of *fun* and *pks* clusters.

Since we identified polyketide synthase (PKS) genes on both pVP-R2lc01 and pVP-R2lc02, we used the secondary metabolite prediction software antiSMASH to identify potential clusters. This analysis revealed that each pVP-R2lc plasmid was predicted to carry a single PKS cluster, here referred to as *fun* and *pks* clusters, located on pVP-R2lc01 and pVP-R2lc02, respectively (Fig. 4C).

Further analysis by MultiGeneBlast (30) did not identify a biosynthetic gene cluster (BGC) similar to those found on pVP-R2lc02. However, when we used single protein sequences to search the NCBI Swiss-Prot database, we identified homologs (27 to 63% amino acid identity) that have been biochemically characterized (Table S3). Nonredundant searches revealed putative homologs in Lactococcus lactis subsp. *cremoris* KW10. Although this gene cluster in KW10 is not intact and separated in different contigs, we identified that all 15 genes in the *pks* cluster have a homolog in the KW10 genome (Table S4).

**FIG 5:**
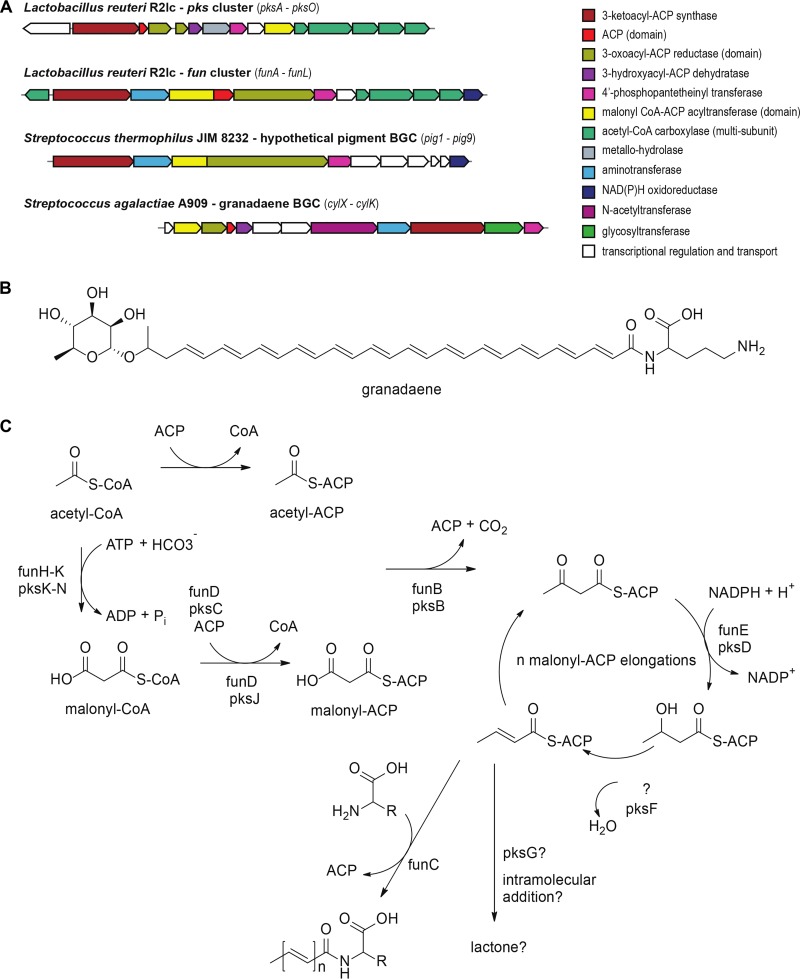
*In silico* analyses of the *fun* and *pks* clusters. (A) Schematic representation of *Lactobacillus* and *Streptococcus* type II polyketide-like fatty acid BGCs. Genes are color-coded by predicted function. (B) Chemical structure of granadaene, produced by group B *Streptococcus*. The polyene chain is formed by 13 elongation rounds, conjugated to the nonproteinogenic amino acid ornithine, and terminally glycosylated with rhamnose. (C) Comparison of the proposed biosynthetic pathways and predicted compounds for *fun* and *pks* clusters.

MultiGeneBlast analyses revealed two gene clusters homologous to the *fun* cluster one in Leuconostoc gasicomitatum LMG 18811 (LEGAS_1823 to LEGAS_1830) and another in Streptococcus thermophilus JIM 8232 (*pig-1* to *pig-9*) (Fig. 5A). The latter BGC is predicted to be involved in group B *Streptococcus* pigment biosynthesis (31). These types of pigments, known as granadaene, have been structurally characterized for Streptococcus agalactiae A909 (Fig. 5B) (32) and *Propionibacterium* (33, 34).

The final product chain length (number of extensions) cannot be predicted from the L. reuteri R2lc-derived PKS clusters, although BLAST searches of the *fun* and *pks* clusters identified enzymes related to long-chain fatty acid machinery and short-chain fatty acid machinery, respectively. Both BGCs have a dedicated multisubunit acetyl-CoA carboxylase (*funH* to *funK* [*funH-K*] and *pksI-N*) for the biotin-dependent carboxylation of acetyl-CoA to form malonyl-CoA. A malonyl CoA:ACP (acyl carrier protein) acyltransferase (*pksJ* and *funD*) is predicted to transfer the malonate to a dedicated ACP domain in the same polypeptide (*funD*) in the *fun* cluster and is encoded separately (by *pksC*) in the *pks* cluster. A predicted ketosynthase (*funB* and *pksB*) catalyzes the decarboxylative condensation of an acetyl-ACP primer and malonyl-ACP extender units. A 3-oxoacyl-ACP reductase (*funE* and *pksD*) reduces the 3-keto to a 3-hydroxyl group, which is subsequently reduced to an alkene by a 3-hydroxyacyl-ACP dehydratase (*pksF*). Since the *fun* cluster is not predicted to encode a dehydratase, it is plausible that this function is complemented by *pksF* or the regular fatty acid dehydratase. Both BGCs lack an enoyl-ACP reductase for further reduction of the carbon chain, resulting in the conjugated double-bond pattern of the predicted products. The growing unsaturated chain is further extended by iterative cycles incorporating malonyl-ACP, after which both biosynthetic pathways are predicted to diverge. The polyene fatty acid produced by the *fun* cluster is predicted to be condensed to a free l-amino acid by the aminotransferase *funC*, similar to the ornithine conjugation in granadaene. The final product of *pks* is hypothesized to be cyclized to form a lactone. *pksG* encodes a predicted metallo-hydrolase, which is potentially involved in intramolecular addition or lactone cleavage (Fig. 5C). Based on our results following the analyses of the PKS clusters with antiSMASH and MultiGeneBlast software, we conclude that the *fun* and *pks* clusters are predicted to encode type II polyketide/fatty-acid-like biosynthesis machineries.

### The *pks* cluster is responsible for AhR activation in R2lc.

**FIG 6:**
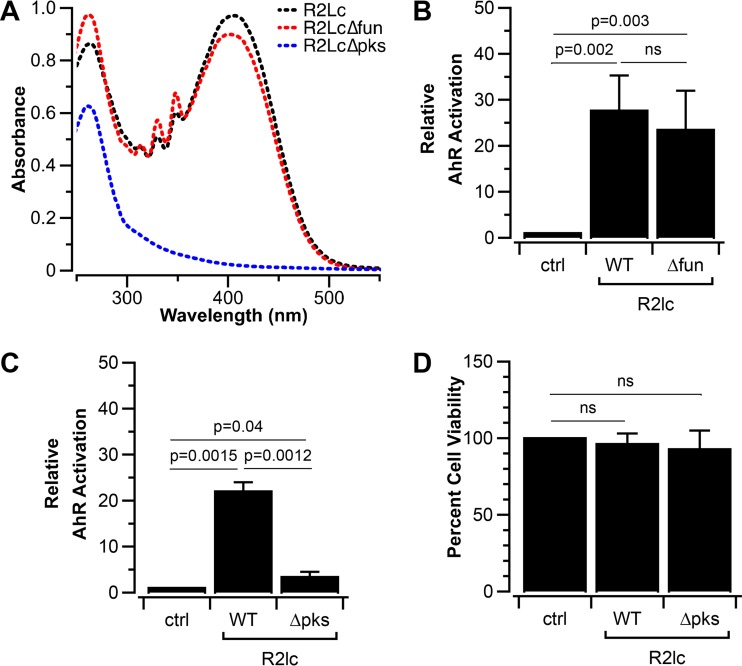
PKS is responsible for AhR activation. (A) R2lc∆*pks* does not absorb at 395 to 410 nm, in the region where R2lc and R2lc∆*fun* exhibited maximum absorbance. (B) The *fun* cluster does not drive AhR activation of L. reuteri R2lc. (C) Inactivation of the *pks* cluster significantly reduces the ability to activate AhR. (D) Extracts used for AhR activation assays do not impact cell viability. Data shown in bar graphs are averages of results from at least 3 biological replicates, and error bars represent standard deviations. A one-sample *t* test was used to compare strains and the negative control, and Student’s *t* test was used to compare wild-type and mutant strains (a *P* value of <0.05 was considered significant). ns, not significant; WT, wild type.

To investigate if the PKS clusters play a role in AhR activation, we generated R2lc derivatives in which the *fun* or *pks* cluster was inactivated. For R2lc Δ*fun*, we deleted approximately 1.4 kb that corresponds to *funE-G*. This region included the gene encoding a 228-amino-acid phosphopantetheinyl transferase (PPTase) protein, which catalyzes the posttranslational modification of acyl carrier proteins in PKS pathways. R2lc Δ*fun*, but not R2lc Δ*pks*, retained its pigmented color, and R2lc *Δfun* exhibited absorption characteristics similar to those of wild-type R2lc (λ_max_ = 405 to 409 nm) (Fig. 6A). Also, R2lc *Δfun* activated AhR similarly to wild-type R2lc (Fig. 6B).

To investigate the role of the *pks* cluster in AhR activation, we replaced the *pksG-I* genes with a single promoter-gene fusion encoding chloramphenicol (Cm) resistance to yield R2lc *Δpks*::Cm. When we assessed the AhR activation potential of R2lc Δ*pks*::Cm, we observed a significantly reduced ability to activate AhR (Fig. 6C), while cell viability upon exposure of the different bacterial supernatants was unaffected (Fig. 6D). In conclusion, the *pks* cluster from pVP-R2lc02 is responsible for the AhR-activating phenotype of L. reuteri R2lc.

### L. reuteri 2010 activates AhR via a predicted gene cluster orthologous to *pks*.

**FIG 7:**
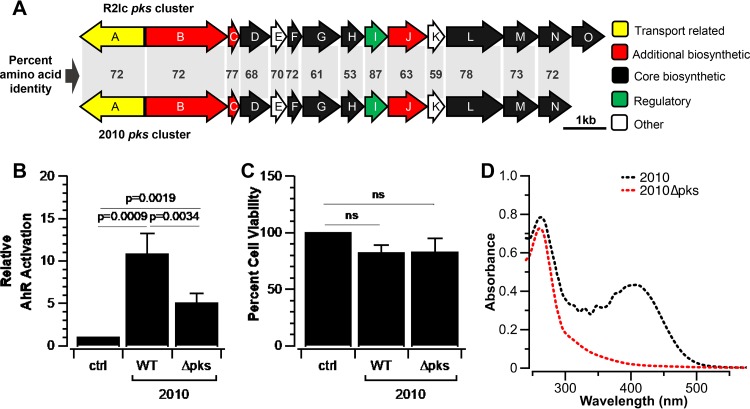
2010 carries a PKS cluster similar to *pks* in R2lc. (A) Percent amino acid identities of homologous genes in *pks* clusters in R2lc and 2010. Transport-related, additional biosynthetic, core biosynthetic, regulatory, and other genes are represented. (B) Deletion of a 1.5-kb region (ORFs *pksG-I*) in 2010 (2010 Δ*pks*) reduces the strain’s ability to activate AhR. (C) Extracts used for AhR activation assays derived from L. reuteri 2010 and L. reuteri 2010 Δ*pks* do not impact cell viability. (D) 2010 shows a maximum absorption band at 405 to 409 nm, which was not observed in 2010 Δ*pks*. Data shown represent averages of results from three biological replicates, and error bars represent standard deviations. A one-sample *t* test was used to compare strains and the negative control, and Student’s *t* test was used to compare wild-type and mutant strains (a *P* value of <0.05 was considered significant). ns, not significant; WT, wild type.

To identify the mechanism by which L. reuteri 2010 activates AhR, we first determined the genome sequence. Since we have identified the pathway by which R2lc activates AhR, we searched the 2010 genome for R2lc *pks* homologs. We identified a homologous gene cluster in 2010 with an identical gene organization (Fig. 7A), which was located on a single 69-kb contig. By inverse PCR, we were not able to find the adjacent contigs, and we did not identify open reading frames that putatively encode a replication-associated protein on the 69-kb contig. Also, unlike the plasmids in R2lc, we did not detect differences in the depth of sequencing coverage, which led us to suggest that the L. reuteri 2010 *pks* cluster could be encoded from the chromosome. Compared to R2lc *pks*, the 2010 *pks* cluster shares considerable identity on the amino acid level, ranging from 53% to 87%. Not surprisingly, the antiSMASH analysis predicted that this cluster encodes an aryl polyene (APE) biosynthetic synthase cluster. To test if the *pks* cluster is responsible for AhR activation of L. reuteri 2010, we deleted the *pksG-I* region, followed by an AhR activation assay. Indeed, 2010 Δ*pks* had a significantly reduced AhR activation capacity compared to the wild type (Fig. 7B). Also, here, the reduced AhR activation capacity was not linked to differences in cell viability, since supernatants derived from L. reuteri 2010 wild-type and 2010 Δ*pks* strains yielded comparable cell survival patterns (Fig. 7C). Similarly, 2010 Δ*pks* is not pigmented and did not show an absorption band at 405 to 409 nm (Fig. 7D).

## DISCUSSION

In this study, our aim was to gain an understanding of the potential of Lactobacillus reuteri to activate AhR at the species level. We screened a library of L. reuteri strains, derived from different hosts, and identified two orange-pigmented strains of rodent origin that are potent activators of AhR. By genome sequencing, comparative genomics, and targeted gene deletion, we identified that an orthologous PKS cluster is responsible for the AhR-activating phenotype.

In L. reuteri 100-23, the aromatic amino acid aminotransferase (AraT) has been shown to play a key role in AhR activation by metabolizing dietary tryptophan (Trp) into AhR ligands (15). In our model, inactivation of the R2lc strain’s *araT* gene, whose gene product has 96% amino acid homology to AraT of 100-23, did not alter the AhR-activating phenotype compared to the wild-type strain. One possibility is that the effect of R2lc AraT was masked by the robust PKS-mediated AhR activation. However, the double mutant (R2lc Δ*araT* Δ*pks*) revealed an AhR activation phenotype similar to that of R2lc Δ*pks* (data not shown). Thus, AraT does not seem to contribute to AhR activation in our experimental model. Although Trp is present in Dulbecco’s modified Eagle’s medium (DMEM), the rate of metabolism, for example, driven by reduced *araT* gene expression compared to *in vivo* conditions, may not yield enough ligand to robustly activate AhR.

The fact that nearly all L. reuteri isolates show basal AhR activation leads us to hypothesize that select metabolic end products may result in AhR activation. This is somewhat substantiated by our finding that other lactic acid bacteria, including Lactobacillus plantarum and Lactobacillus casei, also yielded a basal level of AhR activation (data not shown). A link between bacterial metabolic end products and AhR activation would open new avenues of research to exploit a combination of probiotics and prebiotics to enhance the basal AhR activation potential, an intriguing idea, especially given the role of AhR in ameliorating intestinal inflammation (35).

We identified PKS clusters as the main driver of AhR activation in L. reuteri R2lc and 2010. PKS clusters are not commonly identified in L. reuteri; the only example described in the literature so far is reutericyclin, a chromosomally encoded secondary metabolite from sourdough isolates (10). Reutericyclin has bacteriostatic and bactericidal activities (36) and is proposed to provide these strains with an ecological advantage (37). The reutericyclin producer L. reuteri strains have been used in animal trials without any adverse effects (38). However, in our AhR assay, supernatants of reutericyclin producer strains L. reuteri TMW1.112 and TMW1.656 were toxic to hepatoma cells; thus, we were not able to assess the potential of these PKS-encoding sourdough isolates to activate AhR, and it was outside the scope of this work to test if the toxicity was driven by reutericyclin.

We identified that the *pks* cluster in R2lc has no homology with any BGCs in the NCBI database. The *fun* cluster has homology with the granadaene pigment pathway in *Streptococcus thermophilus* JIM 8232. Granadaenes and aryl polyenes (APEs) are similar from a biosynthetic point of view (related to fatty acid biosynthesis). Since there is some functional redundancy between both PKS clusters, they could cross talk with each other. For example, the *fun* cluster does not seem to have a 3-hydroxyacyl-ACP dehydratase to reduce the 3-hydroxyl groups in the growing chain to alkenes. This could be done by *pksE* or potentially by borrowing the equivalent enzyme from the fatty acid biosynthesis machinery. Therefore, at this point, we cannot rule out the possibility that R2lc Δ*fun* retained activity due to cross talk between the *fun* and *pks* clusters, and this will be analyzed by future liquid chromatography-mass spectrometry (LC-MS) analyses. However, the fact that the inactivation of *pks* in L. reuteri 2010, a strain that does not carry a *fun* cluster homolog, yielded a phenotype similar to what we observed in R2lc Δ*pks* supports the conclusion that the orthologous *pks* clusters in R2lc and 2010 are responsible for AhR activation.

In conclusion, we identified novel PKS gene clusters in L. reuteri that activate the aryl hydrocarbon receptor. Future studies will focus on the role of L. reuteri PKS in immunomodulation and ecology. Recent work has shown that AhR plays an important role in microbiota establishment in mice. AhR contributes to a selective pressure on the gut microbiota subphylum taxonomic groups, potentially by inducing IL-22-mediated antimicrobial production (39). This, combined with the fact that R2lc is a competitive strain in cocolonization studies with other L. reuteri isolates (21), could indicate a role for AhR in providing R2lc with a competitive advantage in host colonization; however, a direct antimicrobial effect, like reutericyclin, cannot be excluded. Aside from a potential ecological role, the secondary metabolite could contribute to amelioration of intestinal inflammation (40). While R2lc has been demonstrated to ameliorate colitis, the probiotic mechanism has not been fully uncovered. Therefore, we plan to determine the role of the R2lc *pks* cluster in amelioration of colitis.

## MATERIALS AND METHODS

### Bacterial strains and culture conditions.

All bacterial strains, plasmids, and oligonucleotides used in this study are listed in Table 1, Table 5, and Table 6, respectively. Lactobacillus reuteri strains were cultured in De Man-Rogosa-Sharpe (MRS) medium (Difco, BD Biosciences). Unless stated otherwise, we prepared bacterial cultures as follows. Lactobacilli were incubated at 37°C under hypoxic conditions (5% CO_2_, 2% O_2_). Escherichia coli EC1000 was used as a general cloning host and cultured at 37°C in lysogeny broth (LB; Teknova). Electrocompetent E. coli EC1000 cells were prepared as described previously (41). Electrocompetent L. reuteri cells were prepared as described previously (42), with slight modifications. Briefly, L. reuteri cells were grown to an optical density at 600 nm (OD_600_) of 0.6 and harvested by centrifugation (4°C at 3,200 × *g* for 5 min). Cell pellets were washed twice with wash butter (0.5 M sucrose, 10% [vol/vol] glycerol). If applicable, erythromycin was supplemented at 5 μg/ml for *Lactobacillus* strains and at 300 μg/ml for E. coli EC1000 strains.

**TABLE 5:** Plasmids used in this study^*a*^

Plasmid	Characteristic(s)	Reference
pVPL3002	pORI19 harboring L. reuteri-derived *ddl*F258Y	29
pVPL3669	Em^r^; derivative of vector pVPL3002 in which the L. reuteri R2lc *Δfun* deletion cassette was cloned into the MCS	This study
pVPL3805	Em^r^; derivative of vector pVPL3002 in which the L. reuteri R2lc Δ*pks* deletion cassette was cloned into the MCS	This study
pVPL31010	Em^r^; derivative of vector pVPL3002 in which the L. reuteri 2010 Δ*pks* deletion cassette was cloned into the MCS	This study
pVPL31041	Em^r^; derivative of vector pVPL3002 in which the L. reuteri R2lc *Δpks*::Cm insertion cassette was cloned into the MCS	This study

a pVPL, van Pijkeren Laboratory plasmid identification number; MCS, multiple-cloning site; *ddl*F258Y, a derivative of *ddl* in which mutations are made yielding the amino acid change phenylalanine to tyrosine at position 258 in the d-Ala-d-Ala (Ddl) protein.

**TABLE 6:** Oligonucleotides used in this study^*a*^

Oligonucleotide	Sequence (5′–3′)	Description
oVPL49	ACAATTTCACACAGGAAACAGC	F; insert screening of pVPL3002
oVPL97	CCCCCATTAAGTGCCGAGTGC	R; insert screening of pVPL3002
oVPL187	TACCGAGCTCGAATTCACTGG	R; amplifies the pVPL3002 backbone
oVPL188	ATCCTCTAGAGTCGACCTGC	F; amplifies the pVPL3002 backbone
oVPL1730	TGAACCTCAATGTGCCTAGC	F; amplifies the u/s flanking region of the R2lc *Δfun* deletion cassette
oVPL1731	AATTTAGTTGGGTTATGCTA	R; amplifies the u/s flanking region of the R2lc *Δfun* deletion cassette
oVPL1732	**TTA**AAGGTACTGATAATTTCTATC	F; amplifies the d/s flanking region of the R2lc *Δfun* deletion cassette
oVPL1733	TAGCGGACGTCCTGTAAAGT	R; amplifies the d/s flanking region of the R2lc *Δfun* deletion cassette
oVPL1734	AAACGACGGCCAGTGAATTCGAGCTCGGTATGAACCTCAATGTGCCTAGCTGGCTTTATA	LCR bridging oligonucleotide to ligate the plasmid backbone + the u/s flanking region of the R2lc *Δfun* deletion cassette
oVPL1735	TTTTCCCAAATAGCATAACCCAACTAAATTAAGGTACTGATAATTTCTATCAGTAAGTCT	LCR bridging oligonucleotide to ligate u/s and d/s flanking regions of the R2lc *Δfun* deletion cassette
oVPL1736	AATATTCTTAACTTTACAGGACGTCCGCTAATCCTCTAGAGTCGACCTGCAGGCATGCAA	LCR bridging oligonucleotide to ligate the d/s flanking region of the R2lc *Δfun* deletion cassette and plasmid backbone
oVPL1737	CGCTATTACGCCAGCTGGCG	F; sequencing of the R2lc *Δfun* deletion region
oVPL1738	TCTGCTGATGGGCCTATAAAT	R; sequencing of the R2lc *Δfun* deletion region
oVPL1739	TCGCTGCAAAGAGCAATCT	F; DCO PCR screening for R2lc *Δfun*
oVPL1740	GGTGATAAAGTCTTGGCTGGAG	R; DCO PCR screening for R2lc *Δfun*
oVPL2334	AAATATCTCCATGTCCTGGCAATAC	F; amplifies the u/s flanking region of the R2lc *Δpks* deletion cassette
oVPL2335	TATCCCGACGAGCAAGTAAAG	R; amplifies the u/s flanking region of the R2lc *Δpks* deletion cassette
oVPL2336	AATGGGGCTGTTATCGTTTTCC	F; amplifies the d/s flanking region of the R2lc *Δpks* deletion cassette
oVPL2337	AAGCTGTATGGCAGGGCTTTC	R; amplifies the d/s flanking region of the R2lc *Δpks* deletion cassette
oVPL2338	ACATTTAACCTTTACTTGCTCGTCGGGATAATCCTCTAGAGTCGACCTGCAGGCATGCAA	LCR bridging oligonucleotide to ligate the plasmid backbone and u/s flanking region of the R2lc *Δpks* deletion cassette
oVPL2339	AAACGACGGCCAGTGAATTCGAGCTCGGTAAATGGGGCTGTTATCGTTTTCCTGTTTTCT	LCR bridging oligonucleotide to ligate the d/s flanking region of the R2lc *Δpks* deletion cassette and the plasmid backbone
oVPL2340	TCTCCTAAAGAAAGCCCTGCCATACAGCTTAAATATCTCCATGTCCTGGCAATACTAGGT	LCR bridging oligonucleotide to ligate u/s and d/s flanking regions of the R2lc *Δpks* deletion cassette
oVPL2341	TGTCCTAGCTGATGCTGCAAC	F; DCO PCR screening for R2lc *Δpks*
oVPL2342	AATAGTTCCAGGGGTGCTTC	R; DCO PCR screening for R2lc *Δpks*
oVPL2518	TGAAAGTGAGTTGTATGGGTGG	F; amplifies the u/s flanking region of the 2010 Δ*pks* deletion cassette
oVPL2519	TCTAGTTCTCTATAATAATTTACGCGC	R; amplifies the u/s flanking region of the 2010 Δ*pks* deletion cassette
oVPL2520	AACTGTTGGATTTCTTGAAAGTCC	F; amplifies the d/s flanking region of the 2010 Δ*pks* deletion cassette
oVPL2521	AGTCGGGTATTTAGCGCAAATTG	R; amplifies the d/s flanking region of the 2010 Δ*pks* deletion cassette
oVPL2522	AAAACGACGGCCAGTGAATTCGAGCTCGGTAAACTGTTGGATTTCTTGAAAGTCCATAAA	LCR bridging oligonucleotide to ligate the plasmid backbone and u/s flanking region of the 2010 Δ*pks* deletion cassette
oVPL2523	AAGAAAGGCCACCCATACAACTCACTTTCATCTAGTTCTCTATAATAATTTACGCGCTGA	LCR bridging oligonucleotide to ligate u/s + d/s flanking regions of the 2010 Δ*pks* deletion cassette
oVPL2524	GCTTTTTCAATTTGCGCTAAATACCCGACTATCCTCTAGAGTCGACCTGCAGGCATGCAA	LCR bridging oligonucleotide to ligate the d/s flanking region of the 2010 Δ*pks* deletion cassette with the plasmid backbone
oVPL2525	TCTGAAGTAGGTGACGGTGC	F; sequencing of the 2010 Δ*pks* deletion region
oVPL2526	AATCCAATTGTCCCAGGAGTC	R; sequencing of the 2010 Δ*pks* deletion region
oVPL2527	GCTTTTTGTGCTCCTTGACC	F; DCO PCR screening for 2010 *Δpks*
oVPL2528	TGCCGTTTTCTGAGGTGTCG	R; DCO PCR screening for 2010 *Δpks*
oVPL2856	AGTGTCATGGCGCATTAACG	F; amplifies the Cm gene of the R2lc Δ*pks*::Cm insertion cassette
oVPL2857	TTATAAAAGCCAGTCATTAGGCC	R; amplifies the Cm gene of the R2lc Δ*pks*::Cm insertion cassette
oVPL2858	TCTCCTAAAGAAAGCCCTGCCATACAGCTTTTATAAAAGCCAGTCATTAGGCCTATCTGA	LCR bridging oligonucleotide to ligate the d/s flanking region of the R2lc Δ*pks*::Cm deletion cassette with the Cm gene
oVPL2859	CCCTTTATTCCGTTAATGCGCCATGACACTAAATATCTCCATGTCCTGGCAATACTAGGT	LCR bridging oligonucleotide to ligate the u/s flanking region of the R2lc Δ*pks*::Cm deletion cassette with the Cm gene
oVPL2860	TGGGAAACAATTTCCCCGAAC	Internal PCR screening for the Cm gene
oVPL665	TCCTCACTCAAGTGGTGCTG	F; amplifies the GAPDH gene in R2lc and its mutants; used for qPCR analyses
oVPL666	ACCGAATGCTGGGTTAGTAG	R; amplifies the GAPDH gene in R2lc and its mutants; used for qPCR analyses
oVPL3095	TGGCAAACCTTTTTGTTGTTCTGG	F; amplifies the *funB* gene in R2lc and its mutants; used for qPCR analyses
oVPL3096	TCGCATTAATACCTCCAAATCCG	R; amplifies the *funB* gene in R2lc and its mutants; used for qPCR analyses
oVPL3097	ATGTCAGAATGGGTTTTTGCTGG	F; amplifies the *pksB* gene in R2lc and its mutants; used for qPCR analyses
oVPL3098	TGATAAGCCGTGCCCTAAAATTTC	R; amplifies the *pksB* gene in R2lc and its mutants; used for qPCR analyses
oVPL395	ATGCCAGCTACTAAAAAAGAAATCCTTAG	F; amplifies *araT* in 6475; used for MAMA-PCR
oVPL396	TTAATCCTCCTTATTAATGAAGGCCG	MAMA-PCR oligonucleotide; used for screening L. reuteri 6475 Δ*araT*
oVPL401	ACAAAGATTCTTGGTGGGATTCCGATTGAAGTTGATACTTAAGGCGATGATTTTGTTCTCACACCCGCAAGACTCCAAAG	Lagging-strand oligonucleotide that mutates S150X; when incorporated, yields a silent mutation and in-frame stop codon
oVPL402	GGATTCCGATTGAAGTTGATACTTAA	R; amplifies *araT* in 6475; used for MAMA-PCR

a oVPL, van Pijkeren Laboratory oligonucleotide identification number; F, forward; R, reverse; u/s, upstream; d/s, downstream; qPCR, quantitative PCR. The sequence in boldface type indicates a stop codon.

### Reagents and enzymes.

To amplify DNA fragments for cloning and screening, we used Phusion Hot Start DNA polymerase II (Thermo Scientific) and *Taq* DNA polymerase (Denville Scientific), respectively. We used T4 DNA ligase (Thermo Scientific) for blunt-end ligations. If applicable, we treated purified PCR products with DpnI (Thermo Scientific) to remove the plasmid template DNA. Phosphorylation of double-stranded DNA (dsDNA) fragments was performed with T4 polynucleotide kinase (Thermo Scientific). Ligase cycling reactions (LCRs) were performed as described previously (43).

### Construction of suicide shuttle vectors.

To generate mutant strains in lactobacilli, we used our recently developed counterselection plasmid (pVPL3002) (29). To generate R2lc *Δfun*, R2lc *Δpks*, and 2010 Δ*pks* deletion cassettes, 500 to 1,000 bp of upstream and downstream flanking regions of target genes were amplified by colony PCR. To amplify the flanking sequences, we used oVPL1730-oVPL1731 (upstream; R2lc Δ*fun*), oVPL1732-1733 (downstream; R2lc Δ*fun*), oVPL2334-2335 (upstream; R2lc Δ*pks*), oVPL2336-2337 (downstream; R2lc Δ*pks*), oVPL2518-2519 (upstream; 2010 Δ*pks*), and oVPL2520-2521 (downstream; 2010 Δ*pks*), followed by column purification (GeneJET PCR purification kit; Thermo Scientific).

The pVPL3002 backbone was amplified with oVPL187-oVPL188, followed by column purification (GeneJET PCR purification kit; Thermo Scientific) and DpnI treatment. Column-purified amplicons were quantified (Qubit; Life Technologies). The amplicons were mixed at equimolar quantities (0.25 pmol), followed by phosphorylation, ethanol precipitation, and LCR. Following LCR, DNA was precipitated with pellet paint (Novagen), resuspended in 5 μl sterile water, and transformed (at 2.5 kV, 25 μF, and 200 Ω) (Gene Pulser Xcell; Bio-Rad) into electrocompetent E. coli EC1000 cells. By PCR, we screened for insertion of our target sequences using oligonucleotides that flank the multiple-cloning site (oVPL49-oVPL97). Finally, the integrity of deletion cassettes was determined by Sanger sequencing.

To replace the *pksG-I* genes in the R2lc *pks* cluster with the gene encoding chloramphenicol (Cm) resistance, we used our laboratory stock of L. reuteri 6475::Cm to amplify the Cm gene with the P_HELP_ promoter (oVPL2856-2857). The amplicon was cloned into pVPL3805 (R2lc Δ*pks* deletion cassette) by LCR to yield R2lc Δ*pks*∷Cm, which we named VPL31041.

### Generation of L. reuteri mutant strains by homologous recombination.

Three micrograms of plasmid DNA was electroporated into electrocompetent L. reuteri cells. To identify plasmid integration, colonies were screened by PCR with oligonucleotide mixtures oVPL1739-oVPL1740-oVPL97 (upstream single crossover [SCO]; R2lc *Δfun*), oVPL1739-oVPL1740-oVPL97 (downstream SCO; R2lc *Δfun*), oVPL2341-oVPL2342-oVPL97 (upstream SCO; R2lc Δ*pks*::Cm), oVPL2341-oVPL2342-oVPL49 (downstream SCO; R2lc Δ*pks*::Cm), oVPL2525-oVPL2526-oVPL97 (upstream SCO; 2010 Δ*pks*), and oVPL2525-oVPL2526-oVPL49 (downstream SCO; 2010 Δ*pks*). Following confirmation of SCO, bacterial cells were cultured for two passages in MRS broth without antibiotic selection, and cells were plated on MRS agar plates containing 400 μg/ml vancomycin. Vancomycin-resistant colonies represent cells in which a second homologous recombination took place. To identify cells in which a double-crossover (DCO) event took place, we performed PCR using oligonucleotides oVPL1739-oVPL1740 (R2lc *Δfun*) and oVPL2341-oVPL2342 (R2lc Δ*pks*::Cm). We used Sanger sequencing to verify the integrity of the recombinant strains.

### Construction of L. reuteri 6475 Δ*araT* by recombineering.

The gene encoding AraT in L. reuteri 6475 was inactivated by single-stranded DNA recombineering as described previously (44). Briefly, at OD_600_ values of >0.55 and <0.65, L. reuteri 6475 harboring the recombineering plasmid pJP042 was supplemented with 10 ng/ml induction peptide (MAGNSSNFIHKIKQIFTHR; Peptide2.0 Inc.) for 20 min to induce the expression of RecT. Electrocompetent cells were prepared as described above, and 100 μg of the recombineering oligonucleotide (oVPL401) was transformed accordingly. We used a mismatch amplification mutation assay-PCR (MAMA-PCR) (45) with oligonucleotides oVPL395, oVPL396, and oVPL402 to identify recombinant genotypes, which were confirmed by Sanger sequencing.

### Preparation of cell-free bacterial supernatants for AhR activation assays.

*Lactobacillus* cultures were grown in MRS broth for approximately 16 h and subsequently subcultured in prewarmed MRS broth (0.1%, vol/vol) until the OD_600_ reached 2. Cell pellets were harvested by centrifugation (3,200 × *g* for 5 min) and washed with an equal volume of Dulbecco’s modified Eagle’s medium (DMEM). Cells were resuspended in an equal volume of DMEM harboring 5% (vol/vol) newborn calf serum (NBCS; Life Technologies) and 1% (vol/vol) nonessential amino acids (NEAA; Gibco) and incubated for 18 h under hypoxic conditions (5% CO_2_ and 2% O_2_). To prevent settling of the cells, and to maintain hypoxic conditions throughout the culture, we gently mixed the cells on an orbital shaker (standard orbital shaker; VWR) (orbital speed, 165 rpm). After incubation, the supernatants were collected following centrifugation (3,200 × *g* for 5 min), the pH was adjusted to 7.6 with 500 nM NaOH, and the supernatants were subsequently filter sterilized (0.22 μm, polyvinylidene difluoride [PVDF]; Millipore).

### *In vitro* AhR activation assays.

The murine hepatoma cell line H1L6.1c3 (a gift from Gregory Kennedy) was used as a reporter cell line to determine AhR activation (19). Expression of the firefly luciferase gene is driven by a dioxin response element (DRE). The level of luminescence is a direct indicator of the level of AhR activation. Briefly, H1L6.1c3 cells were resuspended to a concentration of 1 × 10^5^ cells/ml in DMEM containing 10% (vol/vol) NBCS, 1% (vol/vol) NEAA, and a 1% (vol/vol) penicillin-streptomycin mixture (Lonza). Per biological replicate, we seeded two 96-well plates (clear-bottom white polystyrene; Corning) with 2 × 10^4^ cells/well; one plate was used to determine the activation of AhR, while the other plate was used to determine cell viability (see below). After a 24-h incubation (37°C with 5% CO_2_), cells were gently washed with 200 μl phosphate-buffered saline (PBS), after which 200 μl of the cell-free bacterial supernatant was added in quadruplicate. As controls, we included DMEM containing 5% NBCS and 1% NEAA or the same medium supplemented with the AhR ligand 6-formylindolo[3,2-b]carbazole (FICZ) (500 nM). Cells were incubated for 18 h, followed by assessment of AhR activation and cell viability. For AhR activation, cells were gently washed with 200 μl PBS, followed by the addition of 50 μl/well luciferase assay reagent (Bright-Glo luciferase assay; Promega). After a 5-min incubation at room temperature (RT), total luminescence was measured with a luminometer (BD moonlight 3010; BD Biosciences). Fold AhR activation was calculated as the fold increase in luminescence relative to the DMEM control.

### Cell viability.

To determine cell viability, we used the CellTiter-Glo 2.0 assay kit (Promega) that quantifies the amount of ATP present, which indicates the percentage of metabolically active cells. Briefly, a cell culture plate was incubated at room temperature for 30 min. Next, 100 μl/well of the supernatant was removed, followed by the addition of 100 μl cell viability assay reagent. Samples were incubated for 10 min at 22°C. The luminescence level per well was determined as described above. Percent cell viability for each sample was calculated based on the percent luminescence relative to the DMEM control that contained 5% NBCS and 1% NEAA.

### Pigment extraction and UV-visible spectrophotometry.

L. reuteri cell pellets (2 ml; OD_600_ = 2) were washed twice with PBS and resuspended in 2 ml of 90% methanol. The cell suspension was vortexed for 30 s, followed by centrifugation (14,000 × *g* for 2 min) and filter sterilization (0.22 μm, PVDF; Millipore). The filtrate was diluted in methanol and transferred into a 1-cm quartz cell. This solution was used to investigate the absorption profile, with scanning at a wavelength range of 240 to 600 nm on a UV-visible spectrophotometer (Shimadzu UV-1601 PC; Shimadzu Corp., Kyoto, Japan) at RT (20°C to 22°C). For AhR assays, the methanol extract was evaporated at room temperature overnight. The precipitate was resuspended in 2 ml complete DMEM containing 5% NBCS and 1% NEAA, which was subsequently used to determine the AhR activation potential of orange pigment with the AhR reporter cell line as described above.

### Whole-genome sequencing.

Genomic DNAs were prepared with a genomic DNA purification kit (Wizard; Promega), and DNA concentrations were determined using the Qubit dsDNA high-sensitivity assay kit (Life Technologies). Sequencing was performed at the University of Wisconsin—Madison Biotechnology Center. Samples were prepared according to the TruSeq Nano DNA LT library prep kit (Illumina Inc.). Briefly, samples were sheared using a Covaris M220 ultrasonicator (Covaris Inc.) and size selected for an average insert size of 550 bp using solid-phase reversible-immobilization bead-based size exclusion. The quality and quantity of the finished libraries were analyzed using an Agilent DNA1000 chip and a Qubit dsDNA high-sensitivity assay kit. Libraries were standardized to 2 nM. Paired-end, 250-bp sequencing was performed using the Illumina MiSeq sequencer and a MiSeq 500-bp (v2) sequencing cartridge. Images were analyzed using the standard Illumina pipeline (version 1.8.2).

### Comparative genome and bioinformatics analyses.

*De novo* assemblies of sequence reads were performed using the SeqMan NGen software package (DNASTAR version 12.3.1.4) with standard settings. We closed the gaps between contigs that consist of unique genes in R2lc by Sanger sequencing and primer walking. Briefly, we determined adjacent contigs by PCR using oligonucleotides located at the end of contigs. Next, we amplified gaps between contigs by PCR, and these DNA fragments were sequenced. For primer walking, new oligonucleotides were designed, and an approximately 500- to 900-bp gap was closed after each round of Sanger sequencing. Oligonucleotides used for gap closure are listed in Table S1 in the supplemental material. Assembled draft genomes were uploaded to the Joint Genome Institute Integrated Microbial Genomes and Microbes (JGI-IMG) database to perform annotation and genome comparison. We determined unique genes in L. reuteri R2lc and 2010 genomes by comparison against the following L. reuteri genomes: 100-23, 3c6, CF48-3A1, DSM20016, I5007, JCI1112, LP167-67, Lpuph1, mlc3, and SD2112. We next uploaded draft genomes to the antiSMASH tool, a Web-based secondary metabolite prediction software (46), to identify PKS clusters. We determined the percent amino acid identity of PKS gene products in R2lc and 2010 using the basic local alignment search tool for proteins (BLASTP) at the National Center for Biotechnology Information.

### Plasmid copy number determination.

Relative copy numbers of the pVP-R2lc01 and pVP-R2lc02 plasmids were determined by quantitative PCR as described previously (47), with slight modifications. We used the chromosomal gene encoding glyceraldehyde-3-phosphate dehydrogenase (GAPDH) as a reference, which is present as a single copy. Bacterial cultures were grown until the OD_600_ reached 2. Five-hundred-microliter bacterial cultures were pelleted by centrifugation (21,130 × *g* for 2 min). Cell pellets were washed twice with 500 μl sterile water (21,130 × *g* for 2 min) and resuspended in 400 μl sterile water, followed by microwave treatment (1,100 W for 2 min). Suspensions diluted 100-fold were used as the template for PCR analyses. PCR mixtures contained 8 μl of the cell suspension (100-fold diluted), 1 μl (250 nM each) of the primer pair, and 10 μl of SYBR green master mix (Bio-Rad). Primer pairs were designed for the single-copy housekeeping gene GAPDH (oVPL665-666), pVP-R2lc01 (oVPL3095-2096), and pVP-R2lc02 (oVPL3097-2098). The PCR efficiency for each primer pair was determined. The threshold cycle (*C_T_*) values were determined using the CFX96 real-time system (Bio-Rad). Relative copy number analyses were performed using the Pfaffl method (48). A total of three biological replicates were performed.

### Accession number(s).

The DNA sequences corresponding to Lactobacillus reuteri R2lc and 2010 have been deposited in GenBank with accession numbers PTLS00000000 and PUXG00000000, respectively.

## Supplementary Material

Supplemental file 1
