# Supplementary material for: Gut Symbionts Lactobacillus reuteri R2lc and 2010 Encode a Polyketide Synthase Cluster That Activates the Mammalian Aryl Hydrocarbon Receptor
Source: Appl Environ Microbiol. 2019 May 2;85(10):e01661-18. doi: 10.1128/AEM.01661-18 (PMC6498181; doi:10.1128/AEM.01661-18)
Supplement: Supplemental file 1 [file AEM.01661-18-s0001.pdf]

SUPPLEMENTARY MATERIAL

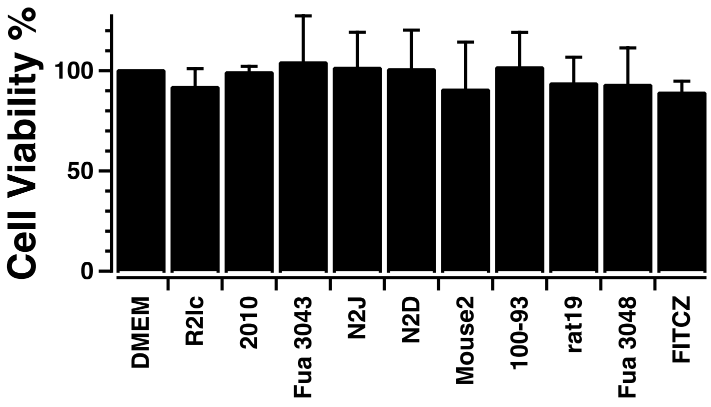

Figure S1: The cell-free bacterial supernatants had no effect on cell viability. Cell viability assay was performed for nine *L. reuteri* strains. Data shown represent averages of three biological replicates and error bars represent standard deviation.

Table S1. Oligonucleotides used for pVP-R2lc02 gap closure.

Oligonucleotide	Sequence (5'-3')
oVPL2319	aagcacccctggaactattg
oVPL2320	acacctgcaacactttgtgaag
oVPL2321	atcgtggagaaatagctgttcg
oVPL2322	agttgggcacccaaaatgtg
oVPL2323	ttgcgcaattgctgatccttg
oVPL2324	tggacctgatctttaatcctcc
oVPL2325	aggcccgttcttgcgtttg
oVPL2326	accgaatgtaggtggaacatc
oVPL2327	tcatcattccacggtatgagatg
oVPL2328	aagtgccgatttagtagctacg
oVPL2329	tcaatatcccaaggcttccag
oVPL2330	ttatgagccgtttgctctgac
oVPL2331	tcctctggtgttcctagcc
oVPL2332	agagaattgcagaaagtggtgac
oVPL2333	tcactagccaatcggggttg
oVPL2344	tgggctgggaccaattattac
oVPL2345	taatgatcgcttcaggtgagg
oVPL2346	ttctcgtgaaccatcctcttc
oVPL2347	tgcaagtttagatagtgggatcg
oVPL2348	ttgccctagcattgatgtgtc
oVPL2373	acccaactccgtctcttcttg
oVPL2374	aagtgcggctcctaatgattg
oVPL2375	catgtggaattgctggtaactta
oVPL2376	accattcagcgattacttaggtc
oVPL2377	taaggggcgtaaatcgtcaag
oVPL2378	agctttatcacttgcaacgcc
oVPL2379	atggaacgccttttggtagtc

oVPL2380	tctgcaaaggactcaccacag
oVPL2381	atgatcaggtccaacgatacg
oVPL2382	tatcccgacgagcaagtaaag
oVPL2383	agcctatttgccaatgtggc
oVPL2384	tatctgggattatagggcgtg
oVPL2385	aggtttagctgctgctcttgg
oVPL2386	agtccggcaatcaaccttctg
oVPL2387	aatttaccggctctgccatttc
oVPL2388	tgggtatgggattattgcagg
oVPL2389	atcaatcagcaggtcaatagcc
oVPL2390	taattgcatacccgcttgtaattg

oVPL: van Pijkeren Lab oligonucleotide identification number.

Table S2. 79 unique genes in R2lc that are not present in other *L. reuteri* strains.

Number	Gene ID	Gene Name	Length (aa)	Located in
1	2717889378	hypothetical protein	106	pVP-R2lc01
2	2717889379	hypothetical protein	219	pVP-R2lc01
3	2717889380	Relaxase/Mobilisation nuclease domain-containing protein	461	pVP-R2lc01
4	2717889381	mobilisation protein (MobC)	124	pVP-R2lc01
5	2717889382	hypothetical protein	78	pVP-R2lc01
6	2717889384	putative transcriptional regulator	100	pVP-R2lc01
7	2717889385	RelE toxin of RelE / RelB toxin-antitoxin system	129	pVP-R2lc01
8	2717889386	hypothetical protein	122	pVP-R2lc01
9	2717889390	Beta-ketoacyl synthase, N-terminal domain	798	pVP-R2lc01
10	2717889391	glycine C-acetyltransferase	399	pVP-R2lc01
11	2717889392	acyl carrier protein	663	pVP-R2lc01
12	2717889393	NAD(P)-dependent dehydrogenase, short-chain alcohol dehydrogenase family	847	pVP-R2lc01
13	2717889394	Phosphopantetheinyl transferase	228	pVP-R2lc01
14	2717889395	transcriptional regulator, TetR family	203	pVP-R2lc01
15	2717889396	acetyl-CoA carboxylase biotin carboxyl carrier protein	135	pVP-R2lc01
16	2717889575	3-oxoacyl-[acyl-carrier-protein] synthase II	698	pVP-R2lc02
17	2717889576	acyl carrier protein	90	pVP-R2lc02
18	2717889578	hypothetical protein	109	pVP-R2lc02
19	2717889580	Glyoxylase, beta-lactamase superfamily II	298	pVP-R2lc02
20	2717889581	4'-phosphopantetheinyl transferase superfamily protein	178	pVP-R2lc02
21	2717889592	hypothetical protein	95	pVP-R2lc02
22	2717889593	hypothetical protein	230	pVP-R2lc02
23	2717889594	Relaxase/Mobilisation nuclease domain-containing protein	470	pVP-R2lc02
24	2717889595	mobilisation protein (MobC)	124	pVP-R2lc02
25	2717889596	hypothetical protein	78	pVP-R2lc02
26	2717889597	hypothetical protein	152	pVP-R2lc02
27	2717889696	hypothetical protein	114	NA
28	2717889703	hypothetical protein	90	NA
29	2717889712	tape measure domain-containing protein	766	NA
30	2717889713	hypothetical protein	34	NA

31	2717889746	Glyoxylase, beta-lactamase superfamily II	198	NA
32	2717889842	hypothetical protein	552	NA
33	2717889976	hypothetical protein	55	NA
34	2717890024	hypothetical protein	27	NA
35	2717890089	hypothetical protein	372	NA
36	2717890101	hypothetical protein	80	NA
37	2717890227	hypothetical protein	29	NA
38	2717890265	hypothetical protein	94	NA
39	2717890708	hypothetical protein	30	NA
40	2717890818	hypothetical protein	53	NA
41	2717890832	Membrane protein involved in the export of O-antigen and teichoic acid	467	NA
42	2717890835	hypothetical protein	377	NA
43	2717890838	Predicted hydrolase, HAD superfamily	655	NA
44	2717890839	UDP-N-acetylglucosamine transferase subunit ALG13	165	NA
45	2717890840	Oligosaccharide biosynthesis protein Alg14 like	149	NA
46	2717891063	hypothetical protein	86	NA
47	2717891073	prevent-host-death family protein	86	NA
48	2717891074	toxin-antitoxin system, toxin component, Txe/YoeB family	86	NA
49	2717891075	hypothetical protein	1147	NA
50	2717891076	hypothetical protein	298	NA
51	2717891078	Helix-turn-helix	211	NA
52	2717891287	hypothetical protein	576	NA
53	2717891289	hypothetical protein	269	NA
54	2717891361	drug resistance transporter, EmrB/QacA subfamily	589	NA
55	2717891362	transcriptional regulator, TetR family	177	NA
56	2717891397	hypothetical protein	127	NA
57	2717891398	hypothetical protein	43	NA
58	2717891402	Helix-turn-helix	71	NA
59	2717891403	hypothetical protein	219	NA
60	2717891404	hypothetical protein	121	NA
61	2717891406	hypothetical protein	50	NA
62	2717891407	hypothetical protein	91	NA
63	2717891417	hypothetical protein	154	NA
64	2717891418	hypothetical protein	165	NA
65	2717891421	hypothetical protein	125	NA
66	2717891422	Methyltransferase domain-containing	144	NA

		protein		
67	2717891423	hypothetical protein	47	NA
68	2717891424	hypothetical protein	73	NA
69	2717891426	Helix-turn-helix	143	NA
70	2717891427	hypothetical protein	44	NA
71	2717891428	hypothetical protein	35	NA
72	2717891430	hypothetical protein	120	NA
73	2717891435	hypothetical protein	248	NA
74	2717891466	hypothetical protein	86	NA
75	2717891467	hypothetical protein	142	NA
76	2717891469	Homeodomain-like domain-containing protein	65	NA
77	2717891480	protein of unknown function (DUF3883)	261	NA
78	2717891486	hypothetical protein	182	NA
79	2717891487	Nuclease-related domain-containing protein	636	NA

Table S3. Homologous genes to *pks* cluster. Amino acid sequences of individual genes in the *pks* cluster were searched in NCBI swissport database.

**R2lc protein**	**Similar protein in NCBI database**	**Organism**	**aa identity %**	**Accession Number**
pksA	Methylenomycin A resistance protein	*Bacillus subtilis* subsp. subtilis str. 168	32	Q00538.1
pksB	Beta-ketoacyl-ACP synthase II	*Vibrio cholerae* O1 biovar El Tor str. N16961	30	Q9KQH9.3
pksC	Phosphodeoxyribomutase	*Streptococcus pyogenes* serotype M1	38	P63927.1
pksD	FabG	*Thermotoga maritima* MSB8	36	Q9X248.1
pksE	Hypothetical protein	-	-	-
pksF	FabZ	*Oceanobacillus iheyensis* HTE831	40	Q8EMB1.1
pksG	Probable metallo-hydrolase YqjP	*Bacillus subtilis* subsp. subtilis str. 168	27	P54553.1
pksH	4'-phosphopantetheinyl transferase	*Pasteurella multocida* subsp. multocida str. Pm70	33	Q9CPD1.1
pksI	Negative transcription regulator PadR	*Bacillus subtilis* subsp. subtilis str. 168	32	P94443.1
pksJ	Polyketide biosynthesis malonyl CoA-acyl carrier protein transacylase BaeC	*Bacillus velezensis* FZB42	33	A7Z4X8.1
pksK	Biotin carboxyl carrier protein of acetyl-CoA carboxylase	*Pseudomonas aeruginosa* PAO1	34	P37799.1
pksL	Biotin carboxylase 1;	*Bacillus subtilis* subsp. subtilis str. 168	53	P49787.3
pksM	Acetyl-coenzyme A carboxylase carboxyl transferase subunit beta	*Lactobacillus reuteri* JCM 1112	63	B2G7K3.1
pksN	Acetyl-coenzyme A carboxylase carboxyl transferase subunit alpha	*Enterococcus faecalis* V583	51	Q830B5.1
pksO	Bifunctional ligase/repressor BirA	*Bacillus subtilis* subsp. subtilis str. 168	29	P0CI75.1

Table S4. *Lactococcus lactis subsp. cremoris* KW10 encodes a homologue gene cluster to the *pks* cluster in R2lc and 2010. Amino acid sequences of individual genes in the *pks* cluster were searched in KW10 genome.

Gene	Homologous gene in KW10	aa identity%	Gene ID
pksA	MFS transporter	53	2744054433
pksB	3-oxoacyl-[acyl-carrier-protein] synthase II	46	2744190884
pksC	acyl carrier protein	63	2744190885
pksD	3-oxoacyl-[acyl-carrier protein] reductase	37	2744190886
pksE	Hypothetical protein	36	2744190887
pksF	3-hydroxyacyl-[acyl-carrier-protein] dehydratase	46	2744190888
pksG	glyoxylase-like metal-dependent hydrolase (beta-lactamase superfamily II)	33	2744190889
pksH	4'-phosphopantetheinyl transferase superfamily protein	31	2744190890
pksI	PadR family transcriptional regulator	51	2744190891
pksJ	[acyl-carrier-protein] S-malonyltransferase	35	2744191699
pksK	biotin carboxyl carrier protein	29	2744191696
pksL	biotin carboxylase /acetyl-CoA carboxylase carboxyltransferase subunit alpha	54	2744191694
pksM	acetyl-CoA carboxylase carboxyltransferase subunit alpha	53	2744191693
pksN	acetyl-CoA carboxylase carboxyltransferase subunit alpha	59	2744191692
pksO	BirA family biotin operon repressor/biotin-[acetyl-CoA-carboxylase] ligase	41	2744192508
